# Non-alcoholic Fatty Liver Disease and Depression: Evidence for Genotype × Environment Interaction in Mexican Americans

**DOI:** 10.3389/fpsyt.2022.936052

**Published:** 2022-07-01

**Authors:** Eron Grant Manusov, Vincent P. Diego, Khalid Sheikh, Sandra Laston, John Blangero, Sarah Williams-Blangero

**Affiliations:** ^1^Department of Human Genetics, The University of Texas Rio Grande Valley, Brownsville, TX, United States; ^2^School of Medicine, South Texas Diabetes and Obesity Institute, The University of Texas Rio Grande Valley, Brownsville, TX, United States; ^3^School of Medicine, The University of Texas Rio Grande Valley, Edinburg, TX, United States

**Keywords:** G × E, liver disease, Mexican Americans, depression, heritability

## Abstract

This study examines the impact of G × E interaction effects on non-alcoholic fatty liver disease (NAFLD) among Mexican Americans in the Rio Grande Valley (RGV) of South Texas. We examined potential G × E interaction using variance components models and likelihood-based statistical inference in the phenotypic expression of NAFLD, including hepatic steatosis and hepatic fibrosis (identified using vibration controlled transient elastography and controlled attenuation parameter measured by the FibroScan Device). We screened for depression using the Beck Depression Inventory-II (BDI-II). We identified significant G × E interactions for hepatic fibrosis × BDI-II. These findings provide evidence that genetic factors interact with depression to influence the expression of hepatic fibrosis.

## Introduction

The Rio Grande Valley (RGV) of South Texas is one of the poorest regions of the United States and experiences significant health disparities. The majority (90%) Mexican American population of the RGV faces disproportionately high rates of obesity (55.5%), diabetes (32.5%), and depression (19%) (1, 2). The prevalence of non-alcoholic fatty liver disease (NAFLD) has risen to a global population high of 25–30%, with significant variation among ethnic groups (3–6). The prevalence of NAFLD in the RGV (40%) mirrors the global NAFLD epidemic. The term NAFLD includes a phenotypic range of entities that can be histologically separated into a non-alcoholic fatty liver with the presence of steatosis in 5% of hepatocytes, without signs of hepatocellular injury (hepatocyte ballooning), and non-alcoholic steatohepatitis (NASH) where there is hepatocellular inflammation and damage with or without fibrosis (3, 7). NAFLD is a significant health concern that can progress to hepatocellular carcinoma (6), increases cardiovascular risk, is associated with higher rates of chronic kidney disease, and is an independent risk factor for system-wide metabolic disease (8, 9).

The pathogenesis of NAFLD results from the accumulation of triglycerides (TGs) in hepatocytes. The sterol regulatory element-binding protein-1 (STEBP-1), activated by insulin and the carbohydrate response element-binding protein (Ch REBP), glucose and fructose, and the peroxisome proliferator active receptor (PPAR) gamma, controls lipogenesis (10–12). As hepatocytes are overwhelmed by TG accumulation, there is a rise in hepatocyte dysfunction due to lipotoxicity, reactive oxygen species, resultant inflammation, DNA damage, and consecutive abnormal cell regeneration and apoptosis. Genetic predisposition, epigenetic changes, anabolic stimuli, adipokine modification, the gut microbiome, and infection influence these pathological processes (3).

We can measure liver health by liver biopsy, magnetic resonance imaging, ultrasound, and vibration-controlled transient elastography (VCTE by FibroScan) (3, 13). Although liver biopsy is considered the “gold standard,” VCTE is accurate and facilitates liver health measurement in community-based healthcare and research settings (14–17). The presence of NAFLD is determined based on VCTE results. The FibroScan quantifies the speed of the shear wave propagated by the ultrasonic wave through the liver. The controlled attenuation parameter (CAP) measures liver ultrasonic attenuation, measuring the degree of steatosis. A CAP of 300 dB/m is an accurate cutoff (PPV 95% CI) and NPV (95% CI) for diagnosing fatty infiltration. Liver stiffness measurements (LSMs) are expressed in kilopascals and accurately measure the level of fibrosis stratified into five groups: ≤8, 8.1–13, 13.1–18, 18.1–23, and >23 kPa.

The presence and severity of NAFLD are associated with depression (1, 18–23). Depression and negative psychological factors may inhibit patients from adhering to the necessary diet and exercise regimen for weight loss and liver health (24–27). Depression adversely impacts the management of chronic diseases by its effect on memory, energy, sense of self-efficacy, and satisfaction with care (20, 28–30). A small study of adolescents found that metabolic syndrome is associated with reduced serotonergic brain activity, possibly contributing to mental illness (31, 32). Recently published information supports the role of inflammation in both depression and NAFLD, finding that both illnesses are correlated (1, 20, 22, 26, 33).

This study examines the impact of G × E interaction effects of NAFLD and depression among Mexican Americans in the RGV of South Texas. We are interested in the role of genetics and the environment in our population, specifically, if there is evidence that genetic factors interact with depression to influence the expression of hepatic fibrosis.

## Materials and Methods

The University of Texas Rio Grande Valley IRB approved the study protocol. All participants provided informed consent prior to participating in the study. We evaluated 279 Mexican American participants recruited from the community in an ongoing genetic study for the presence of obesity, diabetes, hypertension, hyperlipidemia, and depression. Information gathered included biometric data, an assessment of depression (Beck Depression Inventory-II, BDI-II), and VCTE results. Inclusion criteria included age of 18 years or older, residence in the RGV, and having four grandparents who are either Mexican or Mexican American.

The BDI-II was used to assess the degree of depressive symptoms present over a 2-week period (34). The BDI-II assesses the severity of depression and is an acceptable screening instrument for depression when administered in both Spanish and English (35–37). We measured hepatic fibrosis reported as the LSM Youden Index (kPa) and analyzed it as a continuous variable (Echosens, Paris, France) (13, 38, 39). Participants were excluded from elastography if they were pregnant, had an implant, or had a pacemaker. We asked participants to fast for at least 3 h before the exam. The participants lay supine, face-up on the exam table, and fully abducted their right arm. The automatic probe section tool within the device chose the correct probe size (M/XL). Ultrasound conduction gel was applied to the abdomen at the 8th–10th intercostal rib space at the mid-axillary line. Measurements were performed by scanning the right liver lobe through the intercostal space. CAP is an average estimate of ultrasound attenuation at 3–5 MHz (dB/m). LSMs are an average measurement of stiffness at a shear wave frequency of 50 Hz. The results are expressed in kilopascals. In this study, only LSM examinations with at least 10 validated measurements and a success rate of at least 30% were considered reliable. The median value of successful measurements was selected as a representative of the LSM (13, 14, 40, 41).

### Statistical Analysis

We estimated heritabilities (h^2^) and genotype × environment interaction using a variance component approach as implemented in the freely available computer program SOLAR.^1^ Each liver-related phenotype (CAP and kPa) was regressed against age, sex, age-squared, sex-by-age, and sex-by-age-squared, and then the regression residuals derived for each trait were normalized using an inverse normal transformation (42).

### Genotype-by Environment (G × E) Interaction Model for Continuous Environments

The base model—known as the polygenic model—is used to obtain estimates of liver trait heritabilities and as a model reference point upon which complex models can be elaborated. For a sample of related individuals, the polygenic model posits that the phenotypic covariance is decomposable into additive genetic and residual environmental variance components, and that inter-individual covariances will be given strictly by the additive genetic variance weighted by the genetic relatedness coefficient, assuming (for genetic covariance) that the pairwise genetic correlation across environments is unity, and that the additive genetic variance is homogeneous. Under the G × E model, we relax these assumptions by expressing both the additive genetic variance and genetic correlations as continuous functions of a specific environment (e.g., extent of depressive symptoms) to capture any potential interaction between the genetic effects (i.e., the additive genetic variance and/or genetic correlation) and the specific environment. The null hypothesis is that the expression of the aggregate of all genotypes underlying a phenotype (polygenotype) is independent of the specific environment. Rejection of the null hypothesis implies that the genotype–phenotype map for the trait in question depends on a specific environment or is a function of the specific environment. We begin to study the problem of the genotype–phenotype map potentially being dependent on the environment by modeling the G × E interaction variance. The G × E interaction variance is zero if the following two conditions are simultaneously true: (1) homogeneity of the additive genetic variance across environments: σ^2^_*g*1_ = σ^2^_*g*2_ = σ^2^_*g*_, where σ^2^_*g*1_ and σ^2^_*g*2_ are the additive genetic variance in environments 1 and 2, respectively; (2) complete pleiotropy (i.e., the same genes are active across environments) in which the genetic correlation (ρ_*g*_) is one across environments: ρ_*g*_ = 1. There is G × E evidence if either null hypothesis is rejected (43). Rejection of either or both is evidence that the *phenotypic response to the environment* has a genetic basis.

We modeled the genetic variance and cross-environment genetic correlation as functions of depression, where the quantitative measure of depression is given as the total score on the BDI-II. Since it is likely that our focal “environment” is also influenced by genetic factors, we first tested for genetic factors underlying the BDI-II measure of depression, and observed a significant heritability of 0.38 (*p* < 1.0 × 10^–5^). Because we are interested in the purely environmental component of depression, we computed a prediction of the associated genetic values using Best Linear Unbiased Prediction (BLUP) methods. BLUP accounts for additive genetic and environmental covariances among relatives based on known pedigree structure (44). We then subtracted the BLUP genetic values for BDI from the original (BDI-derived) depression variable to get a BLUP-computed depression variable that reflects primarily environmental effects (44). This lattermost variable is the focal (genetically corrected) environment in our G × E model.

For the genetic variance function (and similarly for the environmental variance), we modeled the variance using an exponential function of depression, where the exponential function maintains positivity, which is required of a variance (45) σ^2^_*g*_ = exp [α_*g*_ + γ_*g*_ (BDI)], where α_*g*_ and γ_*g*_ are parameters to be estimated. Taking the natural logarithm of the exponential function, the variance homogeneity null hypothesis holds for a slope-term equal to 0: γ_*g*_ = 0. The genetic correlation was modeled using the exponential decay function of the pairwise differences in BDI scores: ρ_*g*_ = exp [−λ| BDI_*x*_ − BDI_*z*_|] where BDI_*x*_ and BDI_*z*_ are the values of the BDI for any two individuals x and z. The null hypothesis that the genetic correlation is equal to 1 is equivalent to λ = 0 because in this event: ρ_*g*_ = exp [−λ| BDI_*x*_ − BDI_*z*_|] = e^0^ = 1.

We carried out model evaluations and hypothesis testing in two stages. In stage one, we examined if the overall G × E interaction model provided a better fit to the data when compared with the polygenic model by way of a likelihood ratio test (LRT). It is important to note that the polygenic model is fully nested within the G × E interaction model and that relative to the polygenic model, the G × E interaction model has three additional parameters (γ_*g*_, γ_*e*_, and λ; α_*g*_ and α_*e*_ are re-parameterized versions of the variances). The LRT statistic for this comparison is distributed as a 50:50 mixture of Chi-squares with 2 and 3 degrees of freedom (df) (42, 43, 46).

In the second stage, we examine the more specific G × E interaction hypotheses. The full G × E model with all parameters estimated was compared with models when either gamma (γ) or lambda (λ) was constrained to 0 to, respectively, test the hypotheses of additive genetic variance homogeneity and a genetic correlation equal to 1. The distributions of the LRT statistics are, respectively, a Chi-square with 1 df, and a 50:50 mixture of a Chi-square with a point mass at 0 and a Chi-square with 1 df (43, 46). As part of this stage, we determined if each of the three additional parameters in the full G × E interaction model (γ_*g*_, γ_*e*_, and λ) should even be included at all by comparing its maximum likelihood estimate (MLE) to its standard error (SE). A parameter is roughly significant if its MLE is greater than twice its SE based on likelihood theory. Therefore, if a parameter SE was greater than its MLE, we judged that parameter to be statistically unimportant. Further, the additional parameters were formally tested by the tests mentioned above. If any of the three additional parameters were found to have SEs greater than their MLEs and if these were found to be formally insignificant, we then compared a reduced version of the G × E interaction model to the polygenic model, excluding the insignificant parameters.

## Results

The demographic characteristics (age, CAP, kPa, BDI-II) by sex of the cohort are listed in Table 1. There were no significant differences between males and females, as inferred from unpaired *t*-tests assuming unequal variances.

**TABLE 1:** Demographic characteristics of the sample.

Trait	Females (*N* = 205)		Males (*N* = 74)	
		
	Mean	SD	Mean	SD
Age	44.29	15.51	46.04	16.28
CAP	286.61	65.19	290.66	63.14
kPa	6.69	4.50	7.13	4.24
BDI-II	5.67	6.26	4.53	5.92

*All four variables were tested for differences across females and males by an unpaired t-test assuming unequal variance, and it was found that there are no significant differences.*

### Heritability

Individuals with a complete data set (279) were analyzed. As reported in Table 2, we found statistically significant moderate heritability for hepatic fibrosis (h^2^ = 0.37, *p* < 0.01) and steatosis (h^2^ = 0.33, *p* = 0.01). We formally compared the full G × E interaction model to the polygenic model for both kPa and CAP (Table 3).

**TABLE 2:** Heritability analysis of FibroScan variables.

Trait	Heritability	Standard error	Sample size	*P*-value
Hepatic fibrosis (kPa)	0.36	0.14	279	<0.01
Steatosis CAP	0.33	0.14	279	0.01

**TABLE 3:** Testing the full G × E interaction model against the polygenic model.

Trait	Model	ln-likelihood	Chi-square	*P*-value
Hepatic fibrosis (kPa)	Polygenic	−129.94		
	Full G × E	−127.28	5.31	0.07
Steatosis (CAP)	Polygenic	−131.43		
	Full G × E	−130.69	1.48	0.48

It is important to note that full G × E interaction model has three additional parameters of interest compared to the basal polygenic model, one of which has a null hypothesis on the boundary of its permissible parameter space. For this reason, the formal comparison gives a 50:50 mixture of Chi-squares with 2 and 3 df. To ensure best-model selection, we took under consideration that the two “slope” parameters, which allow for genetic and environmental variance heterogeneity, both had SEs larger than their respective MLEs, whereas the MLE for the genetic correlation decay parameter was larger than its SE.

Formal 1 df testing of the genetic and environmental variance heterogeneity parameters showed that they are not significant for both CAP and kPa (Tables 4, 5). On the other hand, for kPa, but not for CAP, the genetic correlation decay parameter, which allows for exponential decay from a null hypothesis of 1, was found to be significant both when compared to the full G × E interaction model and when compared to a reduced G × E interaction model where the genetic and environmental variance slope parameters were constrained to 0. Regarding this lattermost result of a significant genetic correlation decay parameter, the comparison is equivalent to comparing a re-parameterized polygenic model (because the three additional parameters are now constrained to their nulls) to a reduced G × E interaction model where now the only additional parameter is the genetic correlation decay parameter. The environmental-dependence of the genetic correlation across environments suggests that different genes are involved in liver variation conditional upon the depression environment. This evidence confirms that genetic factors interact with depression (the environment) to influence the expression of hepatic fibrosis.

**TABLE 4:** Testing the critical parameters of the full G × E interaction model for CAP.

Model	ln-likelihood	Chi-square	*P*-value
Constrained genetic slope	−131.43	1.48	0.58
Constrained environmental slope	−130.97	0.55	0.46
Constrained genetic correlation decay	−131.43	1.48	0.22
Full G × E interaction model	−130.69	0	0.50

**TABLE 5:** Testing the critical parameters of the full G × E interaction model for kPa.

Model	ln-likelihood	Chi-square	*P*-value
Constrained genetic slope	−127.65	0.75	0.39
Constrained environmental slope	−127.30	0.04	0.83
Constrained genetic correlation decay	−129.37	4.17	0.02
Full G × E interaction model	−127.28		

## Discussion

We investigated the impact of G × E interaction on NAFLD variation in Mexican Americans from the RGV. We determined that both hepatic fibrosis and steatosis were moderately heritable. Our heritability findings are consistent with those of earlier studies [heritability = 0.27 (SE = 0.08) in the Old Order Amish Study, and 0.26 (SE = 0.04) in the Framingham Heart Study population] (47). Cohort-specific estimates of heritability for hepatic steatosis in a Hispanic-American population was estimated at 0.20 (SE 0.07) (48). In a cross-sectional analysis of a cohort of well-characterized 60 pairs of twins adjusted for age, sex, and ethnicity, the heritability of hepatic steatosis was 0.52 (95% confidence interval, 0.31–0.73; *p* < 1.1 × 10^–11^) and the heritability of hepatic fibrosis was 0.5 (95% confidence interval, 0.28–0.72; *p* < 6.1 × 10^–11^) (49). The heritability of steatosis and hepatic fibrosis are similarly confirmed by cross-sectional analyses, which have found that first-degree relatives of patients with advanced hepatic fibrosis exhibit advanced fibrosis themselves at a rate 12 times higher (17.9%) than first degree relatives of those without (1.4%) and 78% of parents with children who have NAFLD exhibit hepatic steatosis (50, 51).

Employing variance component models, likelihood-based statistical inference, and further refinement with the BLUP-computed depression variable, we found that the response of fibrosis to the depression environment is heritable; the G × E interaction variance is significant.

Using a meta-analysis, Xiao et al. demonstrated that patients with non-NASH have a significantly higher prevalence of depression than patients with NAFLD (RR: 2.83, *p* < 0.001) (52). Increased expression of inflammatory cytokines seen in steatohepatitis may explain the NAFLD-depression interaction (53, 54). Growing evidence supports NAFLD as a metabolic companion of psychiatric disorders with common shared inflammatory pathways (55–59). There are increased levels of Interleukin L (IL-17), a proinflammatory cytokine, as well as increased T-helper 1 (TH-1) cells (that produce IL-17) in adult patients with depression as compared to healthy controls (60). The presence of TGF-beta is required for the development of TH-1 cells, which are also elevated in depression, providing further evidence for a relationship between depression and inflammation (61).

The role of IL-17 in depression and NAFLD, as well as comorbid visceral adiposity and atherosclerosis, is also well documented. A strong relationship was found between the IL-17-related chemokine eotaxin and Intimate-Media Thickness (a functional and structural marker of the process that relates to coronary artery disease and NAFLD). The association found between the amount of visceral fat and circulating levels of eotaxin and IMT could reinforce the hypothesis that IL-17, released by the visceral adipose tissue, induces eotaxin secretion via the smooth muscle cells present in the atheromatous vessels (62).

It appears that an inflammatory process mediates many chronic illnesses through upregulation of exclusively pro-inflammatory gene expression. This is consistent with our finding that the genetic correlation function for fibrosis decays away from 1 (i.e., from being fully correlated) with increasing differences in the depression environment between any two given individuals, which indicates that individuals at different ends of the depression “spectrum” may be expressing different sets of genes. Concerning inflammation, this may demonstrate a progression from a more neutral set of genes to a relatively pro-inflammatory set of genes.

### Implications for Research

The findings in this report are based on a relatively small (*n* = 279). Future research will focus on larger sample sizes and may identify other potential interactions. Our focus is on Mexican Americans, and future research will determine if the finding of genotype by environment interaction effects between NAFLD and depression is replicated in other populations.

## Conclusion

We examined potential G × E interaction using variance component models and likelihood-based statistical inference in the phenotypic expression of NAFLD including hepatic steatosis and hepatic fibrosis. We assessed depression (environment) using the BDI-II and identified significant G × E interactions for hepatic fibrosis and depression. These findings provide evidence that genetic factors interact with depression to influence the expression of hepatic fibrosis. Future directions will focus on identifying the nature of the interactions and the specific genes involved.

## Data Availability Statement

The raw data supporting the conclusions of this article will be made available by the authors, without undue reservation.

## Ethics Statement

The studies involving human participants were reviewed and approved by The University of Texas Rio Grande Valley Institutional Review Board. The patients/participants provided their written informed consent to participate in this study.

## Author Contributions

EM, SL, JB, and SW-B contributed to the conception and design of the study. SL organized the database. VD performed the statistical analysis. KS helped write the first draft. All authors contributed to the manuscript, and read and approved the submitted version.

## Conflict of Interest

The authors declare that the research was conducted in the absence of any commercial or financial relationships that could be construed as a potential conflict of interest.

## Publisher’s Note

All claims expressed in this article are solely those of the authors and do not necessarily represent those of their affiliated organizations, or those of the publisher, the editors and the reviewers. Any product that may be evaluated in this article, or claim that may be made by its manufacturer, is not guaranteed or endorsed by the publisher.

## Footnotes

1
http://solar-eclipse-genetics.org/brief-overview.html
